# Recurrence of controlled mycosis fungoides after SARS-CoV-2 infection^☆^

**DOI:** 10.1016/j.abd.2022.06.001

**Published:** 2022-10-12

**Authors:** Éderson Valei Lopes Oliveira, Lígia M. Landell, Cacilda da Silva Souza

**Affiliations:** a Division of Dermatology, Department of Internal Medicine, Ribeirão Preto Faculty of Medicine, Universidade de São Paulo, Ribeirão Preto, SP, Brazil; b Department of Pathology and Forensic Medicine, Ribeirão Preto Faculty of Medicine, Universidade de São Paulo, Ribeirão Preto, SP, Brazil; c Laboratory of Pathology Prof. Dr. Humberto de Queiroz, Ribeirão Preto, SP, Brazil

Dear Editor,

Infection caused by Coronavirus 2 (*Betacoronavirus* genus), responsible for the COVID-19 pandemic, can potentially cause severe acute respiratory syndrome (SARS), affect other organs, and trigger autoimmune events as a result of the cytokine storm.^1^, ^2^ Among cutaneous T-cell lymphomas (CTCLs), mycosis fungoides (MF) and Sézary syndrome (SS) are the most common. MF has an indolent course, slow progression, and rarely attains a cure.^1^, ^3^ The report describes the recurrence of controlled MF after SARS-CoV-2 infection and highlights the potential immunogenic effects of the virus as a trigger in CTCLs.

A 62-year-old woman diagnosed with MF of two years evolution was controlled after phototherapy with 8-methoxypsoralen and Ultraviolet A (PUVA), followed by UVB (narrow band). Two weeks after household contact with COVID-19, she developed a maculopapular rash (Fig. 1). After 15 days, the condition persisted, with mild pruritus in areas with erythema and fine desquamation, where reduction or flattening of the papules ocurred, which developed after two months into parchment-like plaques on the trunk, abdomen and limbs (Fig. 2). There were no mucosal lesions, palpable ganglia in the different assessed chains, and systemic signs/symptoms. Among the requested tests, a positive nasal swab for SARS-CoV-2A by immunofluorescence (detection of CoV-2 nucleoproteins) was observed (COI: 94.40; COI-Cutoff Index < 1: non-reactive); D-dimer elevation (1,876 ng/mL; positive > 500 ng/mL); non-reactive serologies for human T lymphotropic viruses 1 and 2; and chest X-ray within the normal range. Skin histopathology showed lymphocytic exocytosis and cellular atypia in the epidermis (Fig. 3a-b), whereas immunohistochemistry showed the predominance of TCD4 lymphocytes and loss of expression of TCD7 lymphocytes (Fig. 3c-d). Clinical staging was established at Ib (T2bN0M0B0).

**Figure 1:**
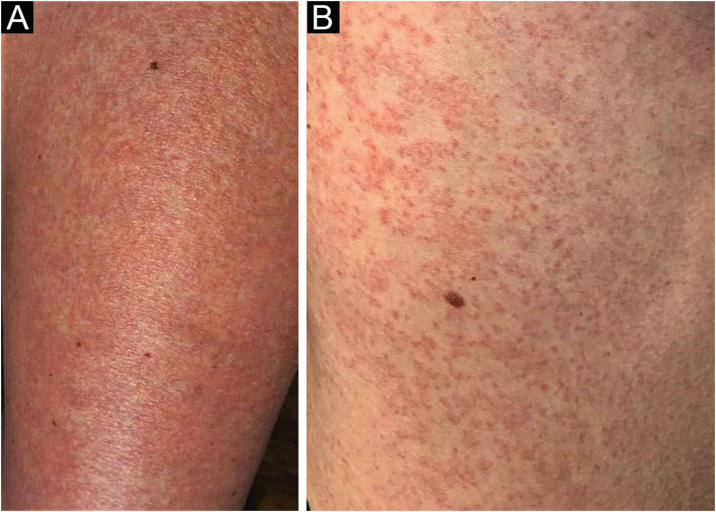
(a) Diffuse erythema and papules on the left thigh; (b) In detail, erythematous-brown follicular papules over erythema on the lateral-posterior aspect of the left trunk.

**Figure 2:**
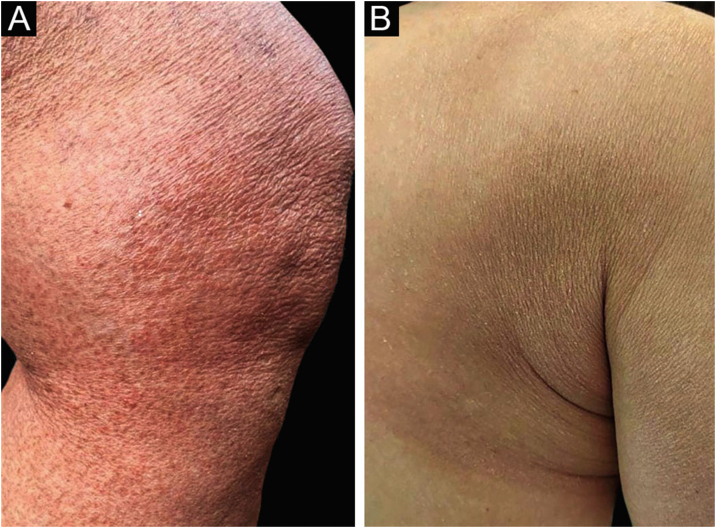
(a) Finely desquamative, parchment-like erythematous-brown plaques on the distal third of the left thigh and knee; (b) and on the right scapular region, after 2 months.

**Figure 3:**
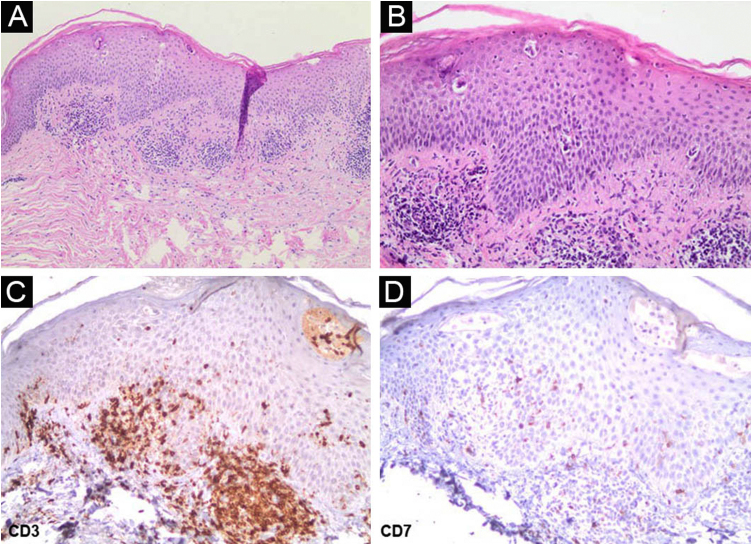
(a) Interstitial and superficial perivascular infiltrate of atypical lymphocytes, with lymphocyte exocytosis (Hematoxylin & eosin, ×100); (b) In detail, atypical cellular infiltrate in the epidermis (Hematoxylin & eosin, ×400); (c) On immunohistochemistry, CD3 positivity in T-lymphocytes (×400); (d) Low expression of CD7 in T-lymphocytes (×400).

The role of environmental factors and infectious agents as triggers or promoters of the development of CTCL is not yet fully established. Moreover, autoimmune disorders can generate a favorable environment for the risk of CTCL. Theories about the pathogenesis of MF and SS include increased Th2 activity, and reduced Th1 activity, the antitumor cytotoxic response of CD8 lymphocytes, the dendritic cell population, and the production of interleukin-12 and interferon-alpha.^2^, ^4^

The immunological dysregulation present in COVID-19 favors the reduction of the functional activity of regulatory T lymphocytes and an imbalance in the production of cytokines, in addition to the elevation of C-reactive protein and D-dimer serum levels.^5^ In addition, the molecular mimicry between SARS-CoV-2 and human proteins favors the production of autoantibodies in genetically predisposed patients, causing exacerbation or emergence of autoimmune/autoinflammatory diseases, such as: Guillain-Barré syndrome, Kawasaki disease, immune thrombocytopenic purpura, antiphospholipid antibodies, thrombosis and, potentially, lupus erythematosus, systemic sclerosis, and pemphigus vulgaris.^4^

Although most patients with indolent or controlled CTCLs are not predisposed to viral infections, conditions at risk for infection and severe symptoms of COVID-19 are considered: aggressive or advanced CTCLs, ongoing immunosuppressive therapy, lymphopenia, chronic organ failure, coexisting comorbidities, advanced age.^2^

This unprecedented report of previously controlled MF, with exuberant and sudden cutaneous recurrence after SARS-CoV-2 infection, indicates viral immunogenic mechanisms as potential triggers of immune dysregulation in CTCLs.

## Financial support

None declared.

## Authors’ contributions

Éderson Valei Lopes de Oliveira: design and planning of the case study; data collection, or analysis and interpretation of data; writing of the article or critical review of the intellectual content; collection, analysis and interpretation of data; intellectual participation in the propaedeutic and/or therapeutic conduct of the studied case; critical review of the literature; approval of the final version of the manuscript.

Ligia Magnani Landell: collection, analysis and interpretation of data; intellectual participation in the propaedeutic conduct of the studied case; approval of the final version of the manuscript.

Cacilda da Silva Souza: design and planning of the study; analysis and interpretation of data; writing of the article or critical review of the intellectual content; collection, analysis and interpretation of data; critical review of the literature; approval of the final version of the manuscript.

## Conflicts of interest

None declared.
